# The Association of *COL1A2* rs17166249 and rs412777 Polymorphisms on the Bone Mineral Density in Polish Postmenopausal Women

**DOI:** 10.3390/biom15060775

**Published:** 2025-05-27

**Authors:** Adam Kamiński, Mateusz Gutowski, Anna Bogacz, Marta Podralska, Izabela Uzar, Michał Soczawa, Maciej Brązert, Bogusław Czerny

**Affiliations:** 1 Department of Children Orthopedics and Musculosceletal Oncology, Pomeranian Medical University in Szczecin, UniiLubelskiej 1, 71-252 Szczecin, Poland; emluc@wp.pl; 2 Department of Anaesthesiology and Intensive Care, Pomeranian Medical University in Szczecin, UniiLubelskiej 1, 71-252 Szczecin, Poland; mateusz.gutowski@pum.edu.pl; 3 Department of Physiology, Poznan University of Medical Sciences, Smoluchowskiego 11, 60-179 Poznan, Poland; 4 Department of Stem Cells and Regenerative Medicine, Institute of Natural Fibres and Medicinal Plants, Kolejowa 2, 62-064 Plewiska, Poland; marta.podralska@iwnirz.pl (M.P.); bczerny@wp.pl (B.C.); 5 Department of General Pharmacology and Pharmacoeconomics, Pomeranian Medical University in Szczecin, 71-230 Szczecin, Poland; uzari@wp.pl; 6 Department and Clinic of Urology and Urological Oncology, Pomeranian Medical University in Szczecin, al. PowstańcówWielkopolskch 72, 70-111 Szczecin, Poland; michal.soczawa@pum.edu.pl; 7 Departmentof Infertility Diagnostics and Treatment, Poznan University of Medical Sciences, Polna 33, 60-535 Poznan, Poland; maciejbrazert@ump.edu.pl

**Keywords:** osteoporosis, polymorphism, *COL1A2*, real-time PCR, bone mineral density

## Abstract

Background: Osteoporosis is a chronic metabolic condition characterized by progressive loss of bone mass and disruption of the bone spatial architecture. Pathological changes are influenced by multiple factors, including genetic predispositions. Identifying risk factors for osteoporosis is crucial for recognizing at-risk populations, implementing preventive strategies, and supporting diagnostics. Type I collagen, composed of two chains—α1(I) and α2(I), encoded by the *COL1A1* and *COL1A2* genes, respectively—plays a key role in the mechanical strength of tissues, including bones. The aim of this study was to assess the effect of the rs17166249 and rs412777 polymorphisms in the *COL1A2* gene on bone mineral density (BMD) in postmenopausal women. Methods: The study included 570 unrelated women: 119 diagnosed with osteoporosis, 96 with osteopenia, and 355 healthy controls. Polymorphisms in the *COL1A2* gene were analyzed using real-time PCR with specific primers and TaqMan probes. Results: The results showed no significant differences in the distribution of genotypes and alleles of rs412777 between the groups. However, the rs17166249 T allele was found to be more prevalent in the osteoporosis group, although the association was not statistically significant after adjusting for confounders. Furthermore, no significant correlations were observed between the genotypes of either SNP and BMD parameters such as T-score, Z-score, and BMD measurements. Conclusion: These findings suggest that while the *COL1A2* gene may have a modest influence on bone health, its role in osteoporosis risk remains inconclusive, highlighting the need for further studies to explore additional genetic and environmental factors.

## 1. Introduction

Osteoporosis is a chronic metabolic condition characterized by progressive loss of bone mass and the disorganization of the spatial structure of bone. Pathological changes result from multiple factors, including hereditary ones. The final value of bone mass is influenced by intrinsic factors (genetic, racial, and sex-related) as well as extrinsic factors (medications taken, chronic diseases) and modifiable factors (diet, physical activity, and nutritional status) [1,2,3].

Studies suggest that certain genetic polymorphisms influence the development of osteoporosis. Polymorphisms in the vitamin D receptor gene, estrogen receptor gene, type I collagen, calcitonin receptor gene, insulin-like growth factor I gene, and the gene-encoding interleukin 6 are strongly correlated with bone mineral density [1,2,3,4,5]. These polymorphisms may accelerate or slow down the changes in bone mineral density, contributing to familial occurrence of this condition.

Collagen is a protein that plays a crucial role in the mechanical strength of tissues such as cartilage, bones, tendons, skin, and sclera (the white part of the eye). The type I collagen molecule consists of two chains: α1(1) and α2(1), encoded by separate genes—*COL1A2* and *COL1A2*. The α1(I) chain, encoded by a gene located at locus 17q21.3-q22, is 18 kbp long. In contrast, the α2(I) chain is encoded by a gene located in locus 7q21.3-q22.4, with a length of 38 kbp.

The structural function of the protein product of *COL1A1* and *COL1A2* genes, which encode the main components of the bone’s organic matrix, explains the frequent and detailed molecular analyses of these alleles and the ongoing search for links between gene structure and susceptibility to bone fractures [6]. Among collagen types I, II, III, V, and IX, which form fibrillar molecules, type I collagen is the primary structural form found in bone tissue [7]. Owing to these properties, this type of collagen is crucial for the mechanical strength of bone. Thus, the genes encoding type I collagen play a significant role in the pathogenesis of osteoporosis.

Nearly two hundred variants concerning the *COL1A1* and *COL1A2* genes have been described. Many of these mutations are responsible for various forms of congenital bone fragility, known as osteogenesis imperfect [8,9,10]. Null mutations in the *COL1A1* gene lead to quantitative defects in collagen production and are usually responsible for the mild form of osteogenesis imperfecta type I. This mild form can also be caused by point mutations in the *COL1A1* gene, such as the substitution of glycine with cysteine at positions 43 and 46 (G>T), or the replacement of arginine with a stop codon (−237C>T) [10,11,12,13]. Structural defects in collagen chains may also result in lethal, severe, or moderate forms of osteogenesis imperfecta. These phenotypes are typically caused by dominant negative missense mutations that disrupt collagen structure [11,12,13].

Nuytinck et al. characterized a point mutation in which glycine is replaced by serine at position 661 of the α1(I) chain of type I collagen in a child with severe congenital bone fragility. Interestingly, the same substitution—glycine for serine at position 661—was previously identified in the α2(I) chain of type I collagen in a postmenopausal woman with osteoporosis [14].

A study by Garnero et al. confirmed that collagen is one of the key factors determining bone mass and quality [15]. It has been shown that *COL1A2* polymorphism is associated with low BMD and increased risk to osteoporotic fractures [16,17,18]. Therefore, the study aimed to investigate the role of two SNPs, rs17166249 and rs412777, in the gene-encoding type I collagen alpha 2 chain in postmenopausal women with low bone mineral density. The rs17166249 variant is located within the 3′ untranslated region (UTR) of the *COL1A2* gene. It was indicated that rs17166249 may influence the binding affinity of microRNAs, such as miR-382, to the COL1A2 mRNA. Specifically, the presence of certain alleles at rs17166249 can alter the interaction between COL1A2 mRNA and miR-382, potentially leading to changes in the expression levels of the COL1A2 protein. These alterations could have implications for bone mineral density and the risk of osteoporotic fractures [17]. However, the exact functional consequences of rs412777 remain unclear, but its association with bone mineral density suggests a potential regulatory role that warrants further investigation.

The aim of this study was to assess the effect of the rs17166249 and rs412777 polymorphisms in the *COL1A2* gene on bone mineral density (BMD) in postmenopausal women.

## 2. Materials and Methods

### 2.1. Study Group

At the Densitometry Laboratory, Clinical Hospital No. 1 of the Pomeranian Medical University in Szczecin, patients were gathered for the study while undergoing bone mineral density testing. Bone mineral density (BMD) was assessed in the lumbar spine, specifically from the L2 to L4 vertebrae, using dual-energy X-ray absorptiometry (DEXA). Measurements were conducted with a LUNAR DPX 100 scanner (Lunar Corp., Madison, WI, USA). To reduce intermachine variability, all patients were scanned using the same DEXA device. Quality control procedures were carried out in accordance with guidelines from the International Society for Clinical Densitometry (ISCD). A total of 570 women provided written consent. Three groups were identified based on the T-score results: The control group had normal bone density, the osteoporosis group had a T-score below −2.5, and the osteopenia group had a T-score between −1 and −2.5. Information on the disease’s presence, medication use, age at first and last menstruation, number of pregnancies, and birth weight was gathered through interviews with each patient. Women who had entered menopause at least a year prior and had not received bone-mass-affecting treatments (such as hormone replacement therapy (HRT), calcitonin, biphosphates, heparin, steroids, thyroid hormones, antiepileptic medications, GnRH analogs, tibolone, or selective estrogen receptor modulators (SERM)) were included in the study. The study excluded patients who had undergone a bilateral ovariectomy, as well as women with autoimmune diseases, connective tissue diseases, neoplastic diseases, hematological diseases, endocrine and metabolic disorders, or kidney diseases, because these conditions may impact bone loss. Written informed consent was obtained from all participants. This study was approved by the Bioethics Committee of the Pomeranian Medical University in Szczecin (No. KB-0012/127/15 of 16 November 2015). The study was conducted in accordance with the Declaration of Helsinki (1975, revised 2000). Informed consent was obtained from all subjects involved in the study.

### 2.2. DNA Isolation and Genotyping

Following the manufacturer’s instructions, genomic DNA was extracted from venous blood using a QIAamp DNA Blood Mini Kit (Qiagen, Hilden, Germany). A spectrophotometer was used to measure the DNA’s purity and concentration, and the purified DNA was stored at −20°C until genotyping was completed. Using the Taqman Probes and real-time PCR (Thermo Fisher Scientific, Waltham, MA, USA), the genotypes of rs17166249 and rs412777 were identified. Then, 1.25 μL of 20 × probe and primers, 12.5 μL of 2 × PCR Master Mix, 11.25 μL of DNA, and DNase-free water were all included in the 25 μL reaction mixture. The following were the conditions for PCR heat cycling: 45 cycles, including denaturation at 95 °C for 15 s and primer annealing at 60 °C, were followed by denaturation for 3 min at 95 °C. The fluorescent signal from the FAM- or VIC-labeled probe was measured for every cycle. A LightCycler 96 device (Roche Diagnostics, Mannheim, Germany) was used for the PCR amplification. The genotyping module of the LightCycler^®^ 96 Software (version 1.2) was then divided into three categories: wild-type, heterozygotes, and mutants. Real-time PCR was performed in duplicates, and both positive and negative controls were included, as well as a no-template control (NTC).

### 2.3. Statistical Methods

Statistical analysis was performed by using R software version 4.3.1 (The R Foundation for Statistical Computing, https://cran.r-project.org, accessed on 20 March 2025). The mean ± standard deviation (SD) or median and interquartile range (IQR, Q1–Q3) were used to summarize normally or non-normally distributed quantitative data, respectively. Measurements between the two groups were compared by student’s t-test or the Wilcoxon rank sum (Mann–Whitney U) test. Pearson’s chi-square (χ^2^) test was used to compare categorical variables. Quantitative measures were compared by ANOVA or Kruskal–Wallis tests, followed by Tukey or Dunn’s tests. Genotype frequency distributions were determined by applying different models of inheritance using the SNPassoc package version 2.1-0 [19]. Haplotype analyses were conducted using Haploview software (version 4.2). For this study, *p* < 0.05 was considered statistically significant.

## 3. Results

### 3.1. Basic Characteristics of the Study Participants

The detailed characteristics of the study population including 570 unrelated women are summarized in Table 1. We divided this sample into three groups according to the T-score values obtained in the DXA scan. Patients with normal bone mineral density (T-score  ≥  − 1.0) were included in the control group (N = 355). Ninety-six patients had osteopenia (T-score: –1 to –2.5), and one hundred and nineteen had osteoporosis (T-score –2.5 or lower). Patients with osteoporosis were statistically significantly older (*p* = 0.014). The mean age in the control group was 53.91 ± 8.69 years, 53.51 ± 8.37 years in the osteopenia group, and 56.45 ± 8.91 years in the osteoporosis group. In the osteoporosis group, women over 60 years of age predominated (36.13%). There was a comparable amount of overweight or obese women in the groups (*p* = 0.312). The majority of women included in the study (85%) gave birth 1 to 7 times in their lives. However, no differences were observed in the number of pregnancies between the groups (*p* = 0.966). Statistically significant differences between groups were also obtained when comparing the results of densitometric parameters (all *p* < 0.001).

### 3.2. Distribution of COL1A2 Genotypes and Alleles in the Studied Groups

The distribution of genotypes for the two *COL1A2* gene variants is presented in Table 2. In the control group, the studied rs17166249 and rs412777 polymorphisms did not deviate from the Hardy–Weinberg equilibrium (χ^2^-test *p* = 0.596 and *p* = 0.718, respectively). No statistically significant difference was detected in the distributions of genotype frequencies of rs412777 between the groups of women with low bone mineral density with controls. The CC, CT, and TT genotypes of the rs17166249 variant were 42.8%, 46.2%, and 11.0% in the controls and 36.5%, 51.0%, and 12.5% in women with osteopenia (*p* = 0.528, in codominant model). When comparing the osteoporosis group with the control group, no significant differences were observed for rs412777. However, for rs17166249, the distribution of the minor T allele in osteoporosis patients was higher than that in the control group (40.8% versus 34.1%, adjusted 1.43 (95%CI: 0.7,2.92), padj. = 0.332). This variant was significantly associated with a 1.35-fold escalating risk of osteoporosis in the log-additive models (OR = 1.35, *p* = 0.057, padj. = 0.315), adjusted with age, birth weight, and age at menarche and at menopause as confounders.

### 3.3. Association Between Genotypes and Alleles with Bone Mineral Density Parameters

The results of parameters obtained in densitometry were analyzed depending on the frequency of genotypes and alleles. The rs412777 genotypes did not show any association with bone mineral density parameters. For rs17166249 genotypes, no statistically significant differences were observed between medians of BMD, BMD%YA, BMD%AM, and Z-score. However, a slight decrease in bone mineral density and BMD and BMD%YA values was observed with the presence of the rarer T allele in patients. The medians of bone mineral density were 1.12 g/cm^3^ [IQR: 0.97;1.25] for the CC genotype, 1.11 g/cm^3^ [IQR: 0.92;1.22] for CT heterozygotes, and 1.09 g/cm^3^ [IQR: 0.90;1.20] for TT (*p* = 0.075). The obtained BMD%YA values were 94% [IQR: 80.00;104.00] for CC, 93% [IQR: 77.00;101.00] for CT, and 91% [IQR: 75.00;100.00] for TT (*p* = 0.074). A statistically significant difference was observed when comparing the median T-score values between the individual genotypes. T-score values were −0.62 [IQR: −1.71;0.38], −0.78 [IQR: −2.30;0.14], and −0.89 [IQR: −2.09;−0.17] for CC, CT, and TT, respectively (*p* = 0.032) (Table 3). Post-hoc analysis showed statistically significant differences between CC vs. TT (*p* = 0.045) and CC vs. CT (*p* = 0.022) genotypes, while differences between CT vs. TT were not significant (*p* = 0.626).

### 3.4. COL1A2 Two-Locus Association with Low Bone Mineral Density

Allele combination frequency was conducted since the study of the two SNPs of the *COL1A2* gene shows that variants rs17166249 and rs412777 are in linkage equilibrium (D’ = 0.165, r2 = 0.008). In all groups, the most common allele combination was CA (40.8% in controls, 39.8% osteopenia and 36.4% women with osteoporosis). A slightly higher incidence of the TA allele combination was observed in women with osteoporosis compared to the control group (frequency 0.296 vs. 0.234 in controls, *p* = 0.063) (Table 4).

## 4. Discussion

The onset of the cessation of ovarian endocrine activity is known as menopause. The majority of women experience menopause between the ages of 45 and 55 as a result of a decrease in estrogen production and a loss of follicular function [20,21]. Natural menopause is considered to have occurred after 12 months of uninterrupted menstrual cessation. As a consequence of estrogen deprivation, osteopenia and osteoporosis in postmenopausal women are common. Numerous studies show that one in ten women globally suffers from osteoporosis, and that 20% of bone loss may happen post-menopause [20,21]. Bone fragility and extremely low bone mineral density (BMD) are characteristics of osteoporosis, which is defined by a decline in the micro- and macro-architecture of bone tissue. A common consequence of postmenopausal osteoporosis is bone fractures, which are linked to excruciating pain, decreased mobility, and functional loss [22,23].

Our findings highlight the multifactorial nature of osteoporosis risk, where both clinical and genetic factors contribute to bone health deterioration in postmenopausal women. Clinically, significant differences were observed between the osteoporosis, osteopenia, and control groups in terms of age, birth weight, age at menarche, menopause timing, and years since menopause—all of which are recognized risk factors influencing bone mineral density. These results reinforce the importance of comprehensive clinical evaluation in identifying individuals at risk for low BMD and fracture.

Nonetheless, both genetics and environmental factors have a significant influence on osteopenia and osteoporosis, which are complex disorders. Numerous studies have shown that changes in the genome—in addition to environmental factors including nutrition, physical activity, sunlight, smoking, the use of oral contraceptives, or vitamin and mineral intakes—influence the development of osteopenia and osteoporosis [24]. It has been established that bone structure maintenance is regulated by several genes. The group of candidate genes that is most frequently studied include genes: *COL1A1*, *COL1A2* (type I collagen), ESR1 and ESR2 (estrogen receptors), *VDR* (vitamin D receptor), as well as *OPG* (osteoprotegerin), *LRP5* (low density lipoprotein receptor-related protein 5), *TGFβ* (transforming growth factor β), STAT1 (signal transducer and activator of transcription 1), *DAAM2* (dishevelled associated activator of morphogenesis 2), and many others [25,26,27,28,29].

In this study, we focused on two polymorphisms in the *COL1A2* gene. This gene encodes the alpha 2 chain of type I collagen, a critical component of the extracellular matrix (ECM). Type I collagen, composed of two alpha 1 chains (encoded by *COL1A1*) and one alpha 2 chain, performs several vital functions. Type I collagen assembles into fibers that form the structural and mechanical scaffold of bone, skin, tendons, cornea, blood vessel walls, and other connective tissues [30,31]. Heterotrimers of two α1(I) and one α2(I) chains are the dominant isoform of type I collagen. However, homotrimers of three α1(I) chains become prevalent in other tissues only in sporadic disorders associated with *COL1A2* deficiency [32,33].

The *COL1A2* gene is located at locus 7q21.3-q22 and contains 52 exons and 51 introns [34]. Variants in this gene are associated with osteogenesis imperfecta (OI) types I–IV, Ehlers–Danlos syndrome type VIIA as well as classical type, Caffey disease, and idiopathic osteoporosis. In Polish patients with osteogenesis imperfecta (OI), mutations in the *COL1A2* gene yielded a variety of phenotypes. Gach et. al. found mutations in “the lethal region” of the *COL1A2* gene among 197 patients [35]. The identified mutations were c.2539G>A (p.Gly847Ser), c.2845G>A (p.Gly949Ser), and c.3215G>T (p.Gly1072Val). Mutation c.2539G>A (p.Gly847Ser) is located in the lethal region of exon 6. This mutation was previously described in three independent studies in patients with mild type I and moderate type IV OI [36,37]. A patient from the Polish cohort with this mutation had moderate deforming type IV OI. The second mutation, c.2845G>A (p.Gly949Ser), is located in the lethal region of exon 7. This mutation was found in two unrelated patients. It was previously described in nine patients with lethal type II, borderline type II/III, and progressively deforming type III OI [37,38,39,40,41]. Both patients in the Polish cohort with this mutation had progressively deforming type III OI. The last mutation, c.3215G>T (p.Gly1072Val), is a novel mutation located in the lethal region of exon 8. The patient with this mutation had mild type I OI. This study showed that, despite being located in lethal regions, these *COL1A2* mutations in Polish patients were associated with non-lethal OI phenotypes [10,35].

The *COL1A2* gene is associated with bone mineral density (BMD), and variations in this gene can influence the risk of osteoporosis and fractures. Several single nucleotide polymorphisms (SNPs) in *COL1A2* have been studied in relation to BMD. It was established that rs42524 polymorphism substitutes alanine for proline and is associated with BMD. The heterozygotes of rs42524 exhibited lower BMD than either homozygote group [16]. Moreover, rs3917, an insertion/deletion polymorphism in the 3′ untranslated region, disrupts the interaction with microRNA-382, and it was proved that it is associated with a reduced risk of osteoporotic fracture [17]. Rare genetic variants in *COL1A2*, p.Gly496Ala and p.Gly703Ser, are associated with low BMD and osteoporotic fractures in Icelanders. Carriers of these polymorphisms do not typically show signs of osteogenesis imperfecta but have lower BMD [18]. On the other hand, Hu et al. showed no association between common genetic variations of *COL1A2* genes and fracture in postmenopausal Chinese women [42].

The aim of this study was to test the impact of two SNPs of COL1A2 on the risk of osteopenia and osteoporosis in postmenopausal women. The first studied SNP, rs412777—also called the PvuII polymorphism—is a single nucleotide polymorphism and has been investigated for its association with various diseases. It is a synonymous variant (c.1446A>C, p.Pro482=) [43]. The rs412777 polymorphism in the *COL1A2* gene is associated with an increased risk of dental fluorosis in the Brazilian population, with the CC genotype associated with a higher risk and the A allele potentially having a protective effect [44]. No such association was observed in Indian and Mexican studies [43,45]. These discrepancies may be due to differences in study populations, fluoride exposure, lifestyle, and other genetic and environmental factors. A meta-analysis examined the association of rs412777 with fracture risk in physically active individuals [46]. Significant heterogeneity and associations with fracture risk were found for the allelic contrast model and the recessive model, but the overall effect was not significant (*p* > 0.05). The study assessed the effect of polymorphisms, including rs412777, on the risk of tendinopathy in Brazilian athletes [47]. The frequency of the minor allele (C) was 36.1% in the control group and 27.8% in the group with tendinopathy. Univariate analysis showed no significant differences in the distribution of rs412777 between the groups with tendinopathy and the control group. Furthermore, rs412777 was one of three SNPs in *COL1A2* (along with rs42524 and rs2621215) used to infer haplotypes. The wild-type haplotype containing the A allele of rs412777 (AGT) had a frequency of 48.4% in controls and 50.9% in tendinopathy cases. Previous studies have shown that polymorphism is associated with variations in bone mineral density. In prepubertal girls, the minor allele was strongly correlated with fracture incidence and variable bone strength parameters [48]. Moreover, Majchrzycki et al. showed that the CC genotype was associated with the lowest body weight (*p* = 0.039) in women with osteopenia. The authors also found a tendency towards lower Z-score values in women with osteoporosis with the AA genotype compared to other genotypes (AA: –4.71 vs. AC: –1.56 and CC: –1.67) [49]. Our investigation of 215 unrelated women with osteopenia and osteoporosis, in contrast to earlier research, found no statistically significant change in the frequency distributions of rs412777 genotypes between the control group and the groups of women with low bone mineral density.

The second SNP under investigation is located in the genome at g.94407235C>T in the noncoding sequence. Several studies have explored the potential association between rs17166249 polymorphisms and susceptibility to reduced bone mineral density (BMD), osteopenia, and osteoporosis. Majchrzycki et al. reported a higher prevalence of the rs17166249 TT genotype among osteoporotic individuals, suggesting its potential involvement in bone fragility pathogenesis and its utility as a genetic marker for osteoporosis risk [49]. In another study, Lindahl et al. examined associations between several *COL1A2* gene polymorphisms and BMD in elderly men [16]. Similarly, Lau et al. assessed *COL1A2* gene polymorphisms in relation to BMD in postmenopausal women, primarily focusing on the PvuII and EcoRI variants [50]. Although rs17166249 was included in the genotypic analysis, the study did not demonstrate any statistically significant association with bone density parameters. Our findings, in contrast to the previous studies presented by Majchrzycki et al., show no statistical difference in the frequency of alleles and genotypes of rs17166249 in relation to osteoporosis [49]. However, there were statistically significant differences when the medians of T-score were compared across genotypes of rs17166249.

In addition, in the present study, allele combination analysis revealed a slightly greater occurrence of the T (rs17166249) and A (rs412777) in women with osteoporosis than in the control group, but this difference was not statistically significant (*p* = 0.063).

Several important limitations of this study should be highlighted. First, the relatively small number of participants may limit the statistical power of the analyses and the generalizability of the results to the broader population of postmenopausal women. Future investigations involving larger and more heterogeneous cohorts are warranted to validate the observed associations and to elucidate the genetic determinants of bone mineral density (BMD) with greater precision. Second, the lack of replication in an independent cohort and the lack of in silico validation limit the reliability and interpretability of the observed associations. Moreover, the study did not include bioinformatic analyses to predict the potential functional consequences of the examined *COL1A2* variants (rs17166249 and rs412777), thereby limiting inferences regarding their biological relevance. Subsequent research should incorporate both experimental and computational approaches to strengthen causal interpretations and clarify the molecular mechanisms involved. A third limitation pertains to the cross-sectional design, which precludes conclusions regarding temporal or causal relationships. Longitudinal studies are needed to assess the dynamic interactions between genetic variation and bone remodeling processes over time.

Finally, it must be noted that functional characterization of the investigated polymorphisms remains limited, and the precise impact of rs17166249 and rs412777 on *COL1A2* gene function has not been clearly established. Additionally, the molecular pathways through which these variants may modulate skeletal metabolism have yet to be delineated. The potential involvement of other, unexamined genetic factors influencing BMD also cannot be excluded, nor can the modulatory effects of environmental or lifestyle factors, including dietary intake, physical activity levels, and hormonal status. Consequently, further research integrating genome-wide data, functional assays, and gene–environment interaction models in large, well-characterized cohorts is essential to comprehensively understand the role of polymorphisms in the pathophysiology of osteoporosis.

## 5. Conclusions

The results from this study suggest that the polymorphisms in the *COL1A2* gene, specifically rs17166249 and rs412777, exhibit limited associations with bone mineral density parameters in women with varying bone health statuses. The analysis revealed a trend towards a higher prevalence of the minor T allele of rs17166249 in women with osteoporosis compared to the control group; however, these differences were not statistically significant after adjusting for potential confounders, including age, birth weight, and reproductive history. Additionally, no significant associations were found between either of the polymorphisms and other densitometric parameters, such as BMD, Z-score, or T-score.

These results imply that while the *COL1A2* gene may play a role in bone metabolism, its influence on BMD and osteoporosis risk appears to be modest and not sufficient to be considered a major determinant of bone health in this population. The lack of robust associations between these genetic variants and BMD underscores the complexity of genetic contributions to osteoporosis, suggesting that additional genetic factors, environmental influences, or gene–environment interactions may also be involved. Future studies with larger cohorts, more precise genotyping methods, and detailed analyses of gene–environment interactions are warranted to further elucidate the role of *COL1A2* and other genetic variants in the pathogenesis of osteoporosis and bone mineral loss.

## Figures and Tables

**Table 1:** Clinical characteristics of the 570 study participants divided according to the T-score.

Variable	Controls (N = 355)	Osteopenia (N = 96)	Osteoporosis (N = 119)	*p*
Age (years)	53.91 ± 8.69	53.51 ± 8.37	56.45 ± 8.91	0.014
Age, n (%) <50 50–59 ≥60	81 (22.82) 192 (54.08) 82 (23.10)	24 (25.00) 57 (59.38) 15 (15.62)	20 (16.81) 56 (47.06) 43 (36.13)	0.009
Birthweight (g)	3623.40 ± 432.60	3152.50 ± 387.34	3041.84 ± 493.72	<0.001
Age at menarche (years)	14.00 [12.00;15.00]	13.00 [11.00;16.00]	12.00 [11.00;14.00]	<0.001
Age at menopause (years)	50.00 [47.50;53.00]	50.00 [47.00;55.00]	49.00 [45.00;50.00]	<0.001
Years since menopause	6.00 [3.00;9.00]	5.00 [2.00;9.00]	10.00 [5.00;14.00]	<0.001
Childbearing years	37.00 [34.00;40.00]	38.00 [34.00;40.00]	36.00 [33.00;39.00]	0.026
Number of pregnancies	2.00 [1.00;3.00]	2.00 [1.00;2.00]	2.00 [1.00;2.00]	0.966
BMI, n (%) <25 ≥25	201 (56.62%) 154 (43.38%)	46 (47.92%) 50 (52.08%)	66 (55.46%) 53 (44.54%)	0.312
BMD L2–L4 (g/cm^2^)	1.21 [1.12;1.27]	0.97 [0.94;1.02]	0.82 [0.79;0.87]	<0.001
L2-L4 YA (%)	101.00 [94.00;106.00]	81.00 [78.00;85.50]	68.00 [66.00;73.00]	<0.001
L2-L4 AM (%)	107.00 [102.00;119.00]	89.00 [84.00;94.00]	77.00 [74.00;82.00]	<0.001
T-score	−0.04 [−0.67;0.57]	−1.90 [−2.17;−1.44]	−3.14 [−3.38;−2.71]	<0.001
Z-score	0.58 [−0.11;1.63]	−0.87 [−1.38;−0.41]	−1.84 [−2.40;−1.17]	<0.001

Normal distributed variables values are exhibited as the means ± SD and medians, and (IQR) is used for non-normal distributed variables values, YA—Young-Adult, AM—Age-Matched.

**Table 2:** Distribution of *COL1A2* genotypes and alleles in studied groups.

SNP	Model	Genotype/Allele	Controls N (%)	Osteopenia N (%)	OR (95%CI)	*p* Crude	*p* adj.	Osteoporosis N (%)	OR (95%CI)	*p* Crude	*p* adj.
**rs17166249**	Codominant	CC	152 (42.8)	35 (36.5)	1.00	0.528	0.564	38 (31.9)	1.00	0.106	0.584
		CT	164 (46.2)	49 (51.0)	1.30 (0.80–2.11)			65 (54.6)	1.59 (1.00–2.50)		
		TT	39 (11.0)	12 (12.5)	1.34 (0.63–2.81)			16 (13.5)	1.64 (0.83–3.25)		
	Log-additive	0,1,2	355 (74.9)	96 (21.3)	1.20 (0.85–1.67)	0.302	0.439	119 (25.1)	1.35 (0.99–1.85)	0.057	**0.315**
	Allele	C	468 (65.9)	119 (62.0)	1.00	0.310	0.446	141 (59.2)	1.00	0.064	**0.332**
		T	242 (34.1)	73 (38.0)	1.19 (0.85,1.65)			97 (40.8)	1.33 (0.98–1.80)		
**rs412777**	Codominant	AA	148 (41.7)	43 (44.8)	1.00	0.563	0.610	50 (42.0)	1.00	0.637	0.509
		AC	160 (45.1)	44 (45.8)	0.95 (0.59–1.52)			57 (47.9)	1.05 (0.68–1.64)		
		CC	47 (13.2)	9 (9.4)	0.66 (0.30–1.45)			12 (10.1)	0.76 (0.37–1.54)		
	Log-additive	0,1,2	355 (74.9)	96 (21.3)	0.86 (0.61–1.20)	0.369	0.322	119 (25.1)	0.93 (0.68–1.26)	0.626	0.825
	Allele	A	456 (64.2)	130 (67.7)	1.00	0.370	0.324	157 (66.0)	1.00	0.627	0.826
		C	254 (35.8)	62 (32.3)	0.86 (0.61,1.2)			81 (34.0)	0.93 (0.68,1.26)		

*p* adj. by age, birth weight, age at menarche and at menopause. Statistically significant values were considered *p* < 0.025 (Bonferroni correction 0.05/2 SNPs).

**Table 3:** *COL1A2* gene rs17166249 and rs412777 variants in relation to DXA scan parameters.

rs17166249	CC (N = 225)	CT (N = 278)	TT (N = 67)	*p*
BMD L2–L4 (g/cm^2^)	1.12 [0.97;1.25]	1.11 [0.92;1.22]	1.09 [0.90;1.20]	0.075
L2–L4 YA (%)	94.00 [81.00;104.00]	93.00 [77.00;101.00]	91.00 [75.00;100.00]	0.074
L2–L4AM (%)	102.00 [91.00;113.50]	100.00 [84.00;108.00]	100.00 [84.00;108.00]	0.090
T-score	−0.62 [−1.71;0.38]	−0.78 [−2.30;0.14]	−0.89 [−2.09;−0.17]	0.032
Z-score	−0.15 [−1.12;0.95]	−0.47 [−1.50;0.76]	−0.71 [−1.55;0.74]	0.246
**rs412777**	**AA (N = 241)**	**AC (N = 261)**	**CC (N = 68)**	
BMD L2–L4 (g/cm^2^)	1.12 [0.92;1.22]	1.11 [0.91;1.22]	1.15 [1.03;1.25]	0.258
L2–L4 YA (%)	93.00 [77.00;101.00]	93.00 [76.00;101.00]	96.00 [85.50;104.00]	0.219
L2–L4AM (%)	100.00 [85.00;109.00]	101.00 [86.00;109.00]	102.00 [92.00;114.00]	0.318
T-score	−0.71 [−2.17;0.11]	−0.79 [−2.13;0.14]	−0.58 [−1.43;0.39]	0.282
Z-score	−0.61 [−1.49;0.56]	−0.15 [−1.46;0.95]	−0.09 [−1.03;1.33]	0.153

*p*-Kruskal–Wallis test.

**Table 4:** The frequency of *COL1A2* allele combination.

COL1A2		Frequency			Controls/Osteopenia		Controls/Osteoporosis	
rs17166249	rs412777	Controls	Osteopenia	Osteoporosis	χ^2^	*p*	χ^2^	*p*
C	A	0.408	0.398	0.364	0.07	0.797	1.37	0.241
C	C	0.251	0.222	0.228	0.69	0.406	0.54	0.461
T	A	0.234	0.280	0.296	1.67	0.197	3.46	0.063
T	C	0.106	0.101	0.107	0.05	0.817	0.08	0.783

## Data Availability

The original contributions presented in this study are included in the article. Further inquiries can be directed to the corresponding author.

## Abbreviations

The following abbreviations are used in this manuscript:

BMD	Bone mineral density
OI	Osteogenesis imperfecta
SNP	Single nucleotide polymorphism
